# Polydnaviruses of Parasitic Wasps: Domestication of Viruses To Act as Gene Delivery Vectors

**DOI:** 10.3390/insects3010091

**Published:** 2012-01-31

**Authors:** Gaelen R. Burke, Michael R. Strand

**Affiliations:** Department of Entomology, The University of Georgia, 120 Cedar St., Athens, GA 30601, USA

**Keywords:** symbiosis, parasitoid, evolution

## Abstract

Symbiosis is a common phenomenon in which associated organisms can cooperate in ways that increase their ability to survive, reproduce, or utilize hostile environments. Here, we discuss polydnavirus symbionts of parasitic wasps. These viruses are novel in two ways: (1) they have become non-autonomous domesticated entities that cannot replicate outside of wasps; and (2) they function as a delivery vector of genes that ensure successful parasitism of host insects that wasps parasitize. In this review we discuss how these novelties may have arisen, which genes are potentially involved, and what the consequences have been for genome evolution.

## 1. Introduction

Beneficial symbiosis is a ubiquitous phenomenon in which two or more organisms live together in a way that improves the fitness of the associated partners. Symbiosis has played a pivotal role in the evolution of major life forms in nature, and is important for the generation of biological diversity [1]. There are many examples of beneficial symbioses between metazoan hosts and microbial symbionts, from deep-sea clams that use bacteria to harvest energy to sap-sucking insects that have nutrition-supplementing bacterial symbionts [2,3]. Microbes such as bacteria and viruses include some of the most diverse lineages of organisms in nature, and due to their ability to live in a wide range of habitats and environments, represent a very large pool of genes that could potentially be used for a host’s benefit. When metazoan/microbial symbioses arise, the genetic material and traits associated with the microbial symbiont are horizontally transferred to the host, making symbiosis an important source for evolutionary innovation. Among the best-studied examples of metazoan/microbial symbioses involve bacterial symbionts of insects [1]. However, insects have also formed associations with other types of microbes including fungi and protozoans [4,5], and viruses as discussed here.

Polydnaviruses (PDVs) that live in association with parasitoid wasps (order Hymenoptera) are the best known example of an insect/viral symbiosis. Parasitoids are insects that are free-living as adults but which lay their eggs in or on the bodies of other insects (the host) where progeny develop by consuming and usually killing the host. The *Polydnaviridae* is a family of double-stranded DNA viruses that is specifically associated with wasps whose hosts are primarily larval stage Lepidoptera (moths and butterflies) (summarized by [6,7]). Wasps inject PDVs into hosts during parasitism, which then express viral gene products that alter host immune defenses, growth and development to optimize conditions for development of the wasp’s offspring.

**Figure 1:**
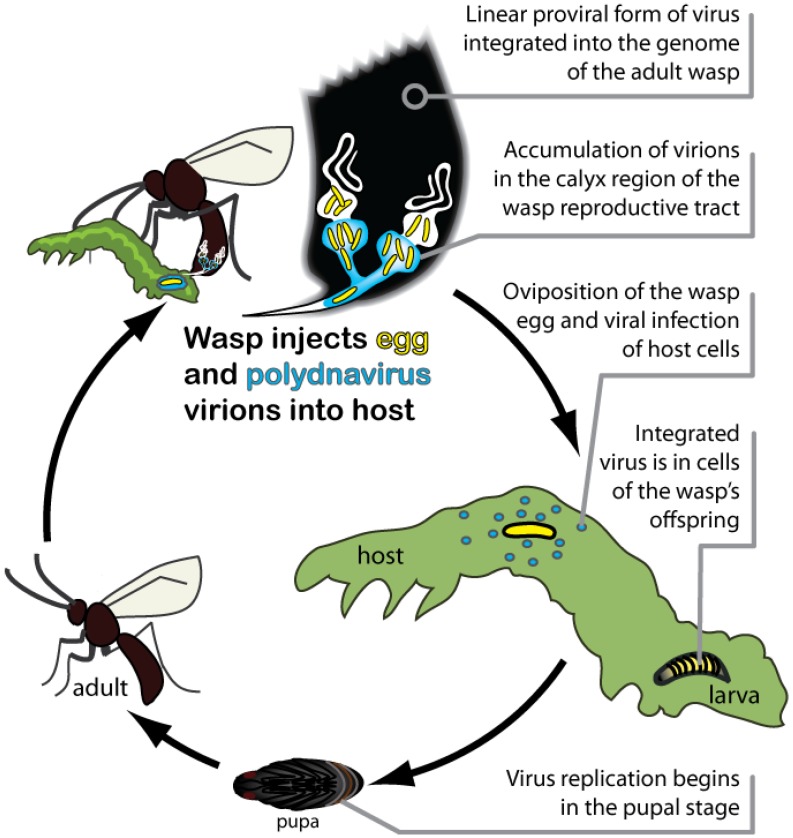
Life cycle of parasitoid wasps and Polydnaviruses (PDVs) parasitizing a lepidopteran larval host.

Each wasp species harbors its own genetically unique PDV, which exists in two forms throughout the wasp life cycle (Figure 1). The proviral form of each PDV genome is integrated into the genome of its associated wasp species, and is transmitted vertically to offspring through the germ line. The encapsidated form of PDV genomes consist of multiple, circular double-stranded DNAs that are packaged into virus particles (virions) during replication. Replication occurs only in the reproductive tract of female wasps in specialized calyx cells (Figure 1). PDVs do not replicate in the wasp’s host, however, because the encapsidated genome lacks the genes required for viral DNA replication and virion production. Since PDVs are replication-defective outside of wasps, viral transmission depends upon the survival of the wasp offspring that carries the PDV genome. Conversely, wasp offspring depend upon the virus for survival in the insect host, making their association mutually beneficial. 

Given that most viruses are parasites, the evolution of a beneficial association between PDVs and wasps is a remarkable innovation [8]. In the first part of this review we summarize key features of this association. We then discuss two questions of central importance in the evolution of PDVs: (1) what changes have PDVs undergone over millions of years in their domestication by wasps; and (2) what modifications have occurred to enable wasps to use PDVs as gene delivery vectors during parasitism?

## 2. PDV Distribution, Origins and Function

### 2.1. PDVs Are Associated with Wasps in Two Families

The order Hymenoptera (wasps, bees and ants) consists of more than 225,000 species that are divided into many families. A majority of these families belong to the suborder Apocrita, which is a monophyletic assemblage that evolved 200–205 million years ago (mya) from an ancestor wasp that was itself a parasitoid [9,10,11]. PDVs are associated with parasitoid wasps in two apocritan families named the Braconidae and Ichneumonidae [12]. In turn, the *Polydnaviridae* is divided into two genera named the *Bracovirus* (BV) and *Ichnovirus* (IV). Phylogenetic studies indicate that the ca. 18,000 species (along with 26,000 estimated undescribed species) of BV-carrying braconids belong to five subfamilies that form a monophyletic group referred to as the Microgastroid complex [13]. This complex diverged approximately 100 mya from the 18 other recognized subfamilies of braconids that do not carry BVs [14]. IVs are associated with only two subfamilies of the Ichneumonidae, the Campopleginae (9000 species) and the Banchinae (4000 species). The phylogenetic relationship of the Campopleginae and Banchinae to one another remains unclear, but no IVs have been observed in any of the other 23 ichneumonid subfamilies that exist worldwide today [7]. 

### 2.2. PDVs Share Several Features

Each PDV from a given wasp species persists during all life stages and in both sexes as an integrated provirus, which is only transmitted to offspring through the germ line (Figure 1). Replication to produce virions occurs only in female wasps and only in the nuclei of specialized calyx cells that form a region of the ovary. In all species studied to date, replication begins during the mid-pupal phase of female wasp development and usually continues during the adult stage [15,16,17,18,19]. Replication of BVs results in the accumulation of large numbers of virions in calyx cell nuclei, which is then followed by cell lysis and release of virions into the lumen of the calyx. In contrast, IVs bud through calyx cells to accumulate in the calyx lumen.

PDVs are so named because the genome packaged into virions during replication consists of multiple circular dsDNA segments that are non-equimolar in abundance. This makes PDVs the only DNA viruses that have multipartite genomes [20]. For BVs, only one genomic segment is packaged per virion, but the capsids of IVs are theoretically large enough to house multiple DNAs [16,21,22]. Segments within IV genomes also undergo a phenomenon called segment nesting, in which progenitor segments give rise to smaller segments by recombination and circularization of parts of the larger segment [7]. 

**Figure 2:**
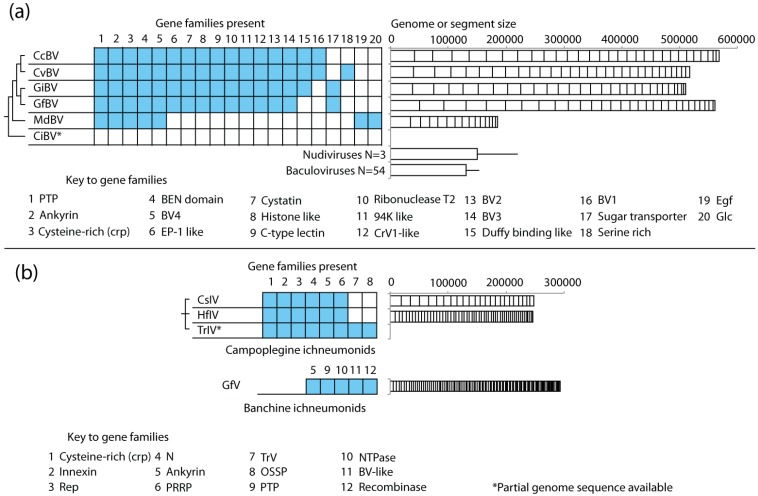
Pictorial summary of conservation of the encapsidated genomes of selected *Bracovirus* (BVs) (**a**) and *Ichnovirus* (IVs) (**b**). Gene families present in each genome are shown in blue boxes on the left side of the figure. The number of genomic segments, their size (kb), and the aggregate size of each genome are shown on the right side of the figure. The size of each genomic segment is represented by vertical bars, which are ordered by size from largest to smallest. BV gene family abbreviations: PTP, protein tyrosine phosphatase; EP-1 like, homologs of “early expressed protein 1” of CcBV; 94K-like, related to baculovirus 94K protein; Egf, epidermal growth factor-like; Glc, glycosylated central domain proteins; CrV1-like, homologs of a gene in CrBV; BV1-4, gene families of unknown function, see [31], IV gene family abbreviations: Rep, repeat element; N, homologs of genes on segment N of CsIV; TrV, homologs of TrV1 from TrIV; PRRP, prolar-residue-rich protein; BV-like, homologs of a CvBV hypothetical protein. The BV genomes shown are *Cotesia congregata* (CcBV) [25], *Cotesia vestalis* (CvBV) [23,28], *Glyptapanteles indiensis* (GiBV) [24], *Glyptapanteles flavicoxis* (GfBV) [24], *Microplitis demolitor* (MdBV) [26], and *Chelonus inanitus* (CiBV) [27]. The IV genomes are *Campoletis sonorensis *(CsIV) [26], *Hyposoter flavicoxis *(HfIV) [30], *Tranosema rostrale *(TrIV) [30], and *Glypta fumiferanae* (GfV) [29]. Some new families were identified in Dupuy *et al. *2011 [31]. In (a) the cladogram of phylogenetic relationships between BVs is derived from Murphy *et al.* 2008 [14]. Also in (a) the average size of known nudivirus and baculovirus genomes is shown.

To date, the encapsidated genomes of 11 PDVs have been sequenced either fully or partially [23,24,25,26,27,28,29,30]. The aggregate size of the genomic DNAs packaged into virions ranges from ca. 180 to more than 600 kb, which makes PDV genomes among the largest viral genomes known (Figure 2). A majority of the predicted genes also form multimember families that are transcribed in parasitized host insects (Figure 2). However, most of these gene families differ between BVs and IVs (Figure 2). Virion morphology also differs with BVs having barrel-shaped capsids surrounded by a single envelope while IVs have fusiform-shaped capsids that are surrounded by two envelopes. Since BV- and IV-carrying wasps are in different families (see above), these results overall indicate that BVs and IVs have different evolutionary origins and that the *Polydnaviridae *is not a natural taxon. These findings also suggest the shared features of PDVs, like genome segmentation, arose by convergent evolution and the similar roles that BVs and IVs play in parasitism.

### 2.3. BVs Evolved from a Nudivirus Ancestor

**Figure 3:**
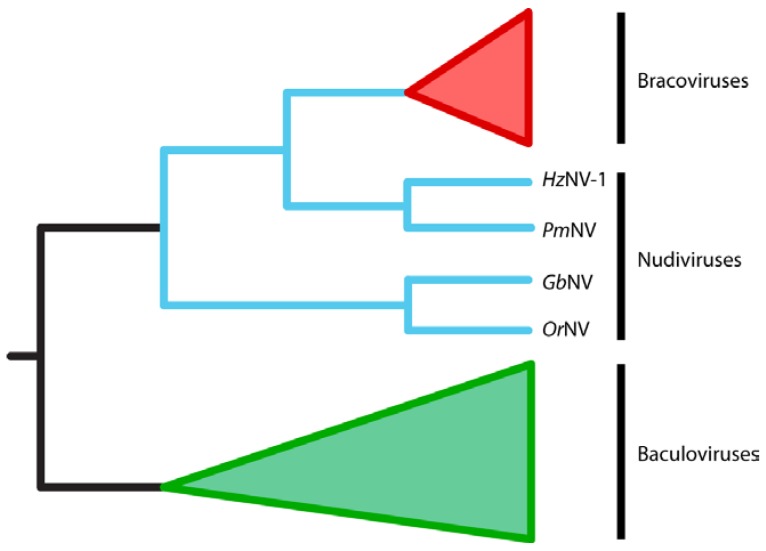
Cladogram showing the evolutionary relationships between bracoviruses (in red), nudiviruses (in blue) and baculoviruses (green). Bracoviruses evolved from within the nudivirus clade and are most closely related to HzNV-1 and PmNV. Nudiviruses and baculoviruses are sister groups of viruses. The figure is adapted from [38].

Early studies of BVs noted that virion morphology was similar to that of viruses in the family *Baculoviridae* while IV virions from campoplegine ichneumonids resembled viruses in the family *Ascoviridae* [32,33,34,35]. However, almost no genes encoded within the encapsidated genomes of BVs or IVs share any significant homology with baculovirus or ascovirus genes. They also do not share homology with any other known viral genes with predicted roles in DNA replication, transcription or virion formation (Figure 2). These results suggested two possibilities for the origins of PDVs: (1) PDVs evolved from a viral ancestor, but the viral genes required for replication are no longer packaged into virions; or (2) PDVs did not evolve from a virus ancestor but instead are the products of wasp genes and possibly a few viral structural genes that were horizontally transferred into the wasp genome [25]. Bèzier *et al.* (2009) [36] sequenced transcripts from the ovaries of two BV-carrying wasps, *Cotesia congregata *and *Chelonus inanitus, *during the pupal stage when replication of *C. congregata *BV (CcBV) and CiBV respectively occurs. The key finding from this study was that several genes strongly upregulated during replication shared significant homology with genes from nudiviruses: a genus first placed in the *Baculoviridae*, but which is now considered a sister taxon ([37,38], Figure 3). These results together with the morphology of virions and monophyly of BV-carrying wasps also argue that BVs evolved from a nudivirus acquired 100 mya by the common ancestor of the microgastroid complex (reviewed in [6,7,14,36]).

The same approach with an IV-carrying campoplegine, *Hyposoter didymator*, failed to identify any genes related to a known virus family [39]. However, some upregulated genes coding for predicted structural proteins in IV virions do have features that are virus-like and form clusters in the wasp genome that are interspersed among wasp genes [39]. Thus, IVs from campoplegine ichneumonids did not evolve from a nudivirus but did likely evolve from another virus that either belongs to an undiscovered taxon or to a virus taxon that has gone extinct. Whether banchine IVs are of the same origin is unclear. 

### 2.4. PDVs Are Essential for Successful Parasitism of Hosts by Wasps

The primary function of PDVs in parasitism is the protection of wasp offspring from the immune defenses of hosts [6,7]. Insects have a well-developed innate immune system for defense against many foreign invaders. Different cell types of the insect immune system produce pattern recognition receptors (PRRs) that recognize pathogen associated molecular pattern (PAMP) molecules associated with different types of infectious organisms. Binding of PAMPs by PRRs stimulates phagocytic and encapsulation responses by immune cells (hemocytes) and activates signaling pathways that regulate the expression of effector molecules like antimicrobial peptides. Recognition of foreign invaders also activates the phenoloxidase (PO) cascade, which consists of multiple proteases that regulate the enzyme PO. PO then catalyzes the formation of cytotoxic intermediates used to produce the pigment melanin [40,41,42,43,44]. 

Parasitoids have evolved a variety of counter strategies to evade or disrupt host immune defenses. Evasion tactics include laying eggs into tissues that are inaccessible to circulating hemocytes or laying eggs that hemocytes do not bind to or recognize as foreign [45,46]. Wasps disrupt host defenses by producing molecules that disrupt hemocyte function or humoral signaling pathways. PDVs are one source of such molecules while another is the venom that wasps also inject into hosts when laying eggs [47]. A third source of immunosuppressive molecules is teratocytes, which develop during embryogenesis of some wasp species, including all BV-carrying braconids, and which are released into the host’s body when wasp eggs hatch [48,49]. PDVs preferentially infect the two major immune tissues of insects (hemocytes and the fat body), and most of the genes in the encapsidated genome are transcribed within 2 h of infection. These gene products then interact to prevent hemocytes from binding to wasp eggs, disable signaling cascades, and inhibit activation of phenoloxidase [50,51,52,53,54]. The origin and diversification of PDV virulence genes is discussed in greater detail below. We refer the interested reader to other recent reviews for details about the function of individual gene products in immunosuppression and other physiological processes [6,7,47,55,56].

## 3. PDVs as Domesticated Extensions of Parasitic Wasps

The preceding summary indicates that PDVs replicate in the reproductive tracts of wasps but do not replicate in the hosts of wasps because their encapsidated genomes lack essential replication genes. However, the expression of several nudivirus-like genes in the reproductive tract of wasps during replication of BVs suggests key genes required for replication are present in the wasp genome. This suggests that the viral genes required for replication have been captured by wasps so that PDVs can no longer propagate themselves in another host. Here we describe how PDVs have been domesticated, and what the consequences of domestication have been for genome evolution.

### 3.1. BVs Likely Evolved from a Pathogenic Nudivirus

While the biology of the IV ancestor remains unknown, the ancestor of BVs was almost certainly a pathogen given that all nudiviruses and baculoviruses are pathogens of insects or related arthropods. Nudiviruses and baculoviruses have large circular dsDNA genomes that persist as episomes and replicate in the nuclei of infected host cells. Both also primarily infect hosts by oral ingestion [37,57]. Baculoviruses usually establish productive infections that are characterized by the coordinated expression of many viral genes, high-level replication of the viral genome, and the release of large numbers of virus particles, that cause the host cell to lyse [57]. Nudiviruses likewise usually establish lytic infections but HzNV-1 and -2 from the moth *Helicoverpa zea* sometimes produce latent, non-lethal infections that are distinguished by a shut off of most genes expressed during a productive infection, formation of few or no virus particles, and persistence of the viral genome as both an episome and integrated provirus [58,59]. Thereafter, latent nudivirus infections can also reactivate to produce a lytic infection [59].

In moths, HzNV-2 localizes to the reproductive tract of both sexes, which causes physiological changes that adversely affect moth reproduction but favor virus transmission [60]. Given the association of some nudivirus isolates with the reproductive system of hosts [37], a plausible scenario for the evolution of BVs is their ancestor established a latent infection in the reproductive tract of a braconid wasp. Unlike HzNV-2, maintenance of the ancestral genome must have initially had little or no fitness cost for the wasp. However, selection thereafter favored alterations that led to the beneficial symbiotic association that exists between BVs and wasps today. So what have these alterations been and how might they have arisen? 

### 3.2. BVs Were Domesticated by Immobilizing Virus Replication Genes in the Wasp Genome

Consider first the consequences of viral genome organization for the replication of BVs, which only occurs in female wasps. BVs package multiple DNAs into virions, but none of these DNAs encode the genes required for particle formation. The location of nudivirus-like BV genes involved in virus particle production in the wasp genome is unclear because no PDV-carrying wasp genomes are sequenced. However, sequencing of BAC clones from wasp genomic libraries provides some insights about BV proviral genome organization. First, data from *C. congregata *show that several BV replication genes of viral origin are dispersed into multiple clusters in the wasp genome. They also are interspersed with wasp genes [36]. Sequencing of BAC clones from the BV-carrying wasps *Glyptapanteles indiensis *and *G. flavicoxis* further show that the proviral genomic segments wasps package into virions exist in tandem arrays that form a total of 6 macroloci [24]. Whether these macroloci are in close or distant proximity to one another in the genome of the wasp is unclear. However, these data do indicate the regions of the proviral genome that are replicated and packaged into virions are separated in the wasp genome from the nudivirus-like replication genes that are required for virion formation. Sequence analysis further indicates the boundaries of the individual genomic segments that are packaged into BV virions each possess a conserved excision/integration motif that contains the tetramer AGCT [24,61,62].

What features of the genome might serve as signals for the packaging into virions of some proviral DNAs but not others? The nudivirus literature itself provides few insights to this question. HzNV-1 is the only nudivirus that has clearly been shown to integrate into the genome of host cells [63]. However, it remains unknown where in the host genome integration occurs, how and where the HzNV-1 genome linearizes when integrating, and what causes the genome to reactivate and replicate.

Assuming the ancestral nudivirus genome integrated as a single large linear DNA, it is possible the excision/integration motifs that flank BV proviral segments originated from the ancestor itself. Thereafter, the nudivirus-like genes still present in BV-carrying wasps most likely lost these flanking excision/integration motifs. This could have occurred by movement of these genes to other locations in the wasp genome. More likely, the integrated ancestral virus genome either duplicated or several copies of the ancestral genome integrated into cells of the wasp followed by elimination of replication genes from some copies and elimination of excision/integration motifs from others [24]. Recent studies also indicate that genomic DNAs in virions from MdBV encode another motif that mediates integration into the genome of host insects by non-homologous recombination (see below). Whether these domains function in wasps is currently unknown but it is possible these elements have also played a role in moving portions of BV genomes within the genomes of wasps.

Regardless of the mechanism(s) involved, the consequence of these events is that some regions of the proviral genome possess flanking motifs the DNA replication machinery of wasps recognize, which results in their selective amplification, excision/circularization, and packaging into virions. In contrast, the ancestral nudivirus genes putatively required for virion formation reside in locations of the wasp genome that appear to lack these motifs. As a result, these genes are expressed during replication in wasp calyx cells but they are not packaged into virions. In turn, virions cannot replicate in the hosts that wasps parasitize, because they lack the genes required to replicate the genome and make virus particles. These changes in the organization of BV genomes are clearly ancient as reflected in the G+C% composition of the nudivirus-like genes, which is more similar to the composition of the coding regions of the wasp genome (34.0%) than the genome of HzNV-1 (45.6%) which is considered the closest currently sequenced nudivirus ancestor of BVs [38,64], Table 1). 

**Table 1:** G+C% content statistics for coding regions of nudivirus-like genes in BVs, HzNV-1 and the *M. demolitor *wasp genome.

Organism and gene set	G+C%	N *
*M. demolitor *transcripts	34.0	66,298 transcripts (32674 loci)
*M. demolitor *nudivirus-like	31.5	133 transcripts (41 loci)
*C. congregata *nudivirus-like	34.0	20
*C. inanitus *nudivirus-like	31.4	18
HzNV *-1 *all coding sequences	45.6	154
HzNV *-1* genes conserved in BVs	47.2	22

* N is the number of transcripts or genes for which the G+C% was calculated and averaged. Some loci in the *Microplitis demolitor *genome are alternatively spliced, while other loci may have been assembled into multiple “transcripts” due to errors of assembly (see [64]).

In the case of IVs, too little is known at present to speculate about the changes they have undergone relative to an ancestor. The limited literature that is available, however, indicates that unlike BVs, at least some of the structural genes required for virion formation are located in clusters that reside in close proximity to the proviral segments that are packaged into virions [39]. It also appears these regions of DNA are amplified during replication but for unknown reasons are not excised and packaged into virions. In summary, PDVs persist as proviruses but differ from other viruses that establish latent infections (bacteriophages, nudiviruses and herpesviruses) because rearrangements of the genome prevent them from producing infectious particles. 

### 3.3. Consequences of Domestication upon Mechanisms of Virus Replication

Comparing the replication genes of BVs to those of related nudiviruses and baculoviruses provides additional insights into innovations that have led to domestication. Little is known about the molecular genetics of nudivirus replication. However, studies of model baculoviruses like *Autographa californica *multiple nucleopolyhedrosis virus (AcMNPV) provide several important insights into the function of key genes (summarized by [57]). Upon entry of the 130 kb AcMNPV genome into the nucleus of a host cell, several viral genes are immediately transcribed by a host RNA polymerase. These ‘early’ genes include single-stranded DNA binding proteins (ssbs), a DNA polymerase, a helicase, and a ligase that are required for viral DNA replication. Other early genes encode the subunits (*p47*, *lef-4*, *lef-8 *and *lef-*9) of a viral RNA polymerase that regulates transcription of ‘late’ and ‘very late’ viral genes. In nudiviruses, an early gene from HzNV-1 (*pag1*) is the only gene transcribed during latency and *hhi1 *is an early gene required for maintenance of active replication [65]. However, no nudivirus *pag1* or *hhi1*-like homologs are known from any baculovirus. Most late and very late genes encode for structural components of the nucleocapsid (*vp39*, *vp1054*, *p6.9 *and *odv*-*ec27*), envelope (*p74*, *pif-1*, *pif-2*, *pif-3*, *19K*, *odv-e56*, *odv-e66*), and under certain conditions the occlusion body (see below). Copies of the circular viral genome are then individually packaged into virions via products of *vlf-1 *and selected other genes [57].

**Figure 4:**
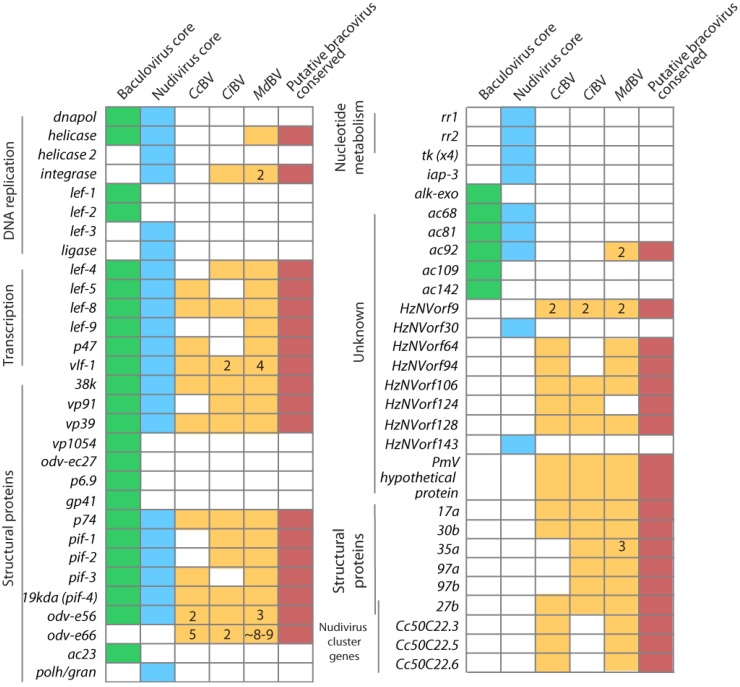
Predicted BV conserved gene set in relation to the core genes for all known baculoviruses and nudiviruses. Green and blue boxes represent core constituents of baculovirus or nudivirus genomes, respectively. Yellow boxes show homologs identified by transcript sequencing of ovaries from three species of braconid wasps. Acronyms are as described for Figure 2. Red boxes show the predicted conserved genes for BVs. Numbers within boxes indicate transcripts for which more than 1 locus was identified (duplicated genes). Adapted from [64].

Sequencing of 54 baculovirus and 4 nudivirus genomes reveals considerable differences in the overall size and total gene content of different isolates. Nonetheless, a set of 30 core genes are present in all baculoviruses of which 20 are present in all nudiviruses [66,67] (Figure 4). Approximately half of these core genes are the aforemetioned early genes whose products regulate viral DNA replication and form the viral RNA polymerase. Most of the other core genes are late and very late genes that code for virion structural components.

How does the core gene set for baculoviruses and nudiviruses compare to BVs? Recent deep sequencing identified most nudivirus-related genes transcribed in ovaries during replication of MdBV in the wasp *Microplitis demolitor *[64] (Figure 4). Together with the aforementioned ESTs generated from *C. congregata *and *C. inanitus* ([36]; see above) we recently suggested a conserved set of genes similar to 35 nudivirus genes for BVs, most of which are are strongly upregulated during replication (Figure 4). Four of these are homologs of nudivirus/baculovirus RNA polymerase subunits, 7 are homologs of nudivirus/baculovirus envelope proteins, 3 encode predicted capsid proteins (*vp39*, *vp91*, *38K*), and 2 encode the late transcription factor *vlf-1* (sometimes in several copies) and putative transcriptional initiation factor (*lef-5*) [64]. Other capsid proteins present in baculovirus genomes (*vp1054*, *p6.9 *and *odv*-*ec27*) but unknown from nudiviruses have not been identified from the ovary transcriptomes of BV-carrying wasps. However, genes similar to 4 predicted structural nudivirus genes (*HzNVorf9*, *64*, *106*, and *PmV hypothetical protein*) [37,67] are present [64,68]. No nudivirus-like *pag1 *and *hhi1 *homologs were identified in the ovary transcriptomes of BV-carrying wasps. The remaining nudiviruses/baculoviruses-like genes in the BV conserved gene set are a helicase, integrase, and *ac92* which encodes a predicated FAD sulfhydryl oxidase that may interact with insect homologs of the tumor suppressor protein p53 [69]. While Wetterwald *et al.* (2010) identified more than 20 non-nudivirus proteins in CiBV virions, genes corresponding to only 6 of these products are conserved among MdBV and CiBV [64,68] (Figure 4). While nudiviruses are the closest relatives of BVs, there is a large amount of amino acid divergence between homologous genes in these groups. This factor, along with the availability of only three complete nudivirus genomes makes the identification of nudivirus-like genes in the wasp genome difficult. Thus, there may be members of the BV conserved gene set that have not yet been identified, because they are highly divergent genes or are not currently represented in sequenced nudivirus genomes.

Taken together, these results suggest that BVs retain a nudivirus-like RNA polymerase and initiation factor (*lef-*5) that likely mediate transcripton of several nudivirus-like structural components that form BV virions. The presence of a nudivirus/baculovirus-like DNA helicase also suggests a small portion of the ancestral viral DNA replication machinery may remain functional. The absence of other viral DNA replication genes, however, argues a key step in the evolution of BVs is that the DNAs packaged into virions are generated by the replication machinery of the wasp. Lastly, several of the nudivirus/baculovirus-like genes present in the genome of BV-carrying wasps are duplicated (Figure 4), which suggests that some ancestral viral genes potentially have evolved novel functions that may or may not involve replication.

### 3.4. Wasps Control PDV Replication

As previously noted, at least some nudiviruses establish latent infections, which in response to unknown factors can be reactivated to produce a high titer of virions. Mobilization of the BV proviral genome to produce virions is likewise a reactivation event. Unlike a latent nudivirus, however, reactivation of BVs is clearly under tight regulatory control by wasps given replication only occurs in one cell type (calyx cells) in females, and synchronously begins in the mid-pupal stage. 

Temporal studies indicate that replication of MdBV in *M. demolitor *begins with transcription of the RNA polymerase subunits which upregulate when female wasp larvae pupate [64]. Soon after, MdBV proviral DNA replication is detected which is then followed by the upregulation of genes coding for virion structural components and the appearance of assembled nucleocapsids in calyx cell nuclei [64]. A similar progression of events occurs during replication of CiBV [70]. These events are also similar to the replication of baculoviruses where expression of early genes like the viral RNA polymerase subunits preceeds viral DNA replication and nucleocapsid assembly [57]. 

Less clear is what signals from the wasp induce the expression of these BV replication genes at pupation or what potentially prevents these genes from being transcribed during other life stages. No homologs have been identfied from BV-carrying wasps for genes like *pag1 *and *hhi1 *which are implicated in regulating latency and replication of nudiviruses. Thus, we think it most likely that the onset of BV replication is regulated by signaling factors of wasp origin. Early studies in the IV-carrying wasp *Campoletis sonorensis *suggested the hormone ecdysone, which regulates the expression of many genes in arthropods, functions as a positive regulator of CsIV replication [7]. Studies have not yet characterized the promoters of BV replication genes to determine whether they contain conserved ecdysone response elements. However, studies in *C. inanitus *indicate that wasp ecdysone titers do not correlate with the timing of CiBV replication, while our own experiments indicate that ecdysteroids do not stimulate the replication of MdBV in *M. demolitor *([70], Burke and Strand, unpublished). Thus, regulation of the timing of replication in IV- and BV-carrying wasps may differ.

### 3.5. Wasp Genes with Potential Roles in Regulating Replication of Viral DNA

Two models have been proposed in the literature for how PDV proviral DNAs are replicated. The first is that replication occurs by locus-specific amplification of the integrated proviral genome in a manner akin to selective amplification of genes by diverse eukaryotes including insects [71]. The second is that a larger progenitor molecule(s) is first excised from the wasp genome and is then amplified by a recombination-dependent or rolling circle mechanism [71,72,73,74]. Subsequent excision and circularization of the genomic DNAs that are packaged into virions is thought to occur by site-specific recombination across direct repeat junctions flanking individual segments [61,62,73]. 

The literature at present does not provide definitive evidence as to which of these models is correct. However, the absence of viral DNA replication genes from the BV core gene set strongly suggests that amplification of proviral DNAs for packaging into virions requires wasp genes. The difficulties in identifying nudivirus-like genes in BVs outlined above are not applicable to the viral DNA replication genes, which, with the exception of *lef-3*, are functionally conserved and present in all complete nudivirus genomes. The absence of a nudivirus/baculovirus-like *dnapol* in the deep sequencing transcriptome data set for *M. demolitor *argues this polymerase activity could be provided by one or more wasp polymerases [64]. All of the standard insect polymerase genes were identified in *M. demolitor *but none exhibited expression profiles that correlated with the onset of MdBV DNA replication [64]. Thus, the wasp DNA polymerase(s) responsible for proviral DNA replication is likely not regulated transcriptionally. Given the importance of insect DNA polymerases in other processes, regulation of proviral DNA replication may depend more on gene products that specifically recruit the appropriate polymerase(s) for this purpose. In contrast, our deep sequencing data set for *M. demolitor *does identify homologs for a network of genes that regulate locus-specific DNA amplification in eukaryotes ranging from *Drosophila melanogaster *to *Tetrahymena* [64]. Moreover, this gene set is strongly upregulated in the wasp ovary in concert with the beginning of proviral DNA amplification. In contrast, no homologs of genes implicated in regulating recombination-dependent or rolling circle amplification by other viruses were identified. Both DNA replication models also depend on a DNA polymerase, but as noted above the identity of this DNA polymerase remains unknown.

### 3.6. Regulation of PDV Virulence Gene Expression and Segment Proliferation in Wasps

Nearly all genes on DNA segments packaged into virions are expressed in the hosts that wasps parasitize. In contrast, PDVs have no pathogenic effects in wasps themselves. It is unknown whether PDV virions are capable of infecting wasp cells, but it is known that most genes of the encapsidated genome are not expressed in wasps at any life stage [75]. The few genes of the encapsidated genome that are expressed also have no known pathogenic functions [75,76,77,78,79,80,81]. These findings thus suggest that PDVs do not cause disease in wasps because the genes responsible are not transcribed. The absence of appropriate activators or divergence of promoter elements between replication genes and genes of the encapsidated genome are the most likely factors that underlie their differential expression in hosts and wasps. Another factor could be that wasp cells produce interfering RNAs as known in *Drosophila*, which repress the expression of parasitic DNA elements by a PIWI-interacting system of interfering RNAs (piRNAs) [82]. 

A final way that wasps control PDVs is the apparent inhibition of genome segment proliferation. While multiple proviral DNA segments are organized into macroloci in the genomes of wasps, wasp genomes do not seem littered with an explosive number of PDV segments. However, given the capability of MdBV genomic segments to rapidly integrate into host cells during parasitism ([62]; see below), it follows that wasps have potentially evolved mechanisms that prevent this from readily occurring in their own genomes. What these mechanisms might be are unknown.

### 3.7. Consequences of Virus Domestication on the Rate of Genome Evolution

**Figure 5:**
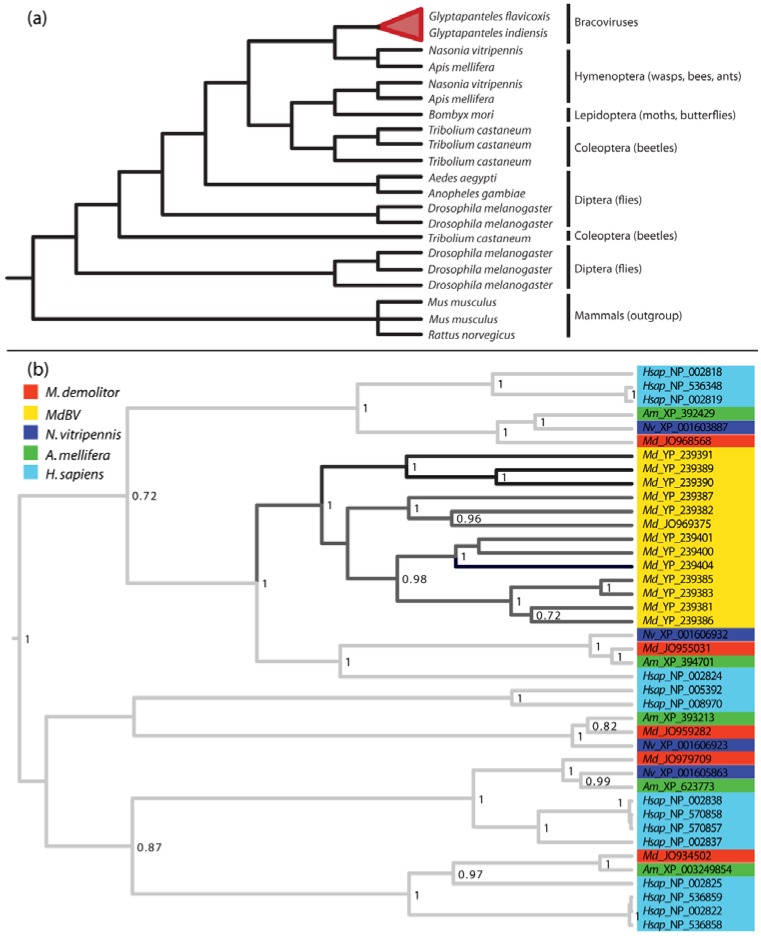
(**a**) Cladogram of the evolutionary relationships between insect and BV Major Facilitator System (MFS) sugar transporter genes. MFS genes from BVs of *G. flavicoxis *and *G. indiensis* clearly evolved from a hymenopteran homolog, and are more closely related to insect genes compared to those from mammals. The tree is adapted from Desjardins *et al.* 2008. [24], (**b**) Phylogenetic reconstruction of the evolution of insect and BV protein tyrosine phosphatase (PTPs). Taxa were chosen from the MdBV genome, *M. demolitor *transcripts, or the *A. mellifera, N. vitripennis, *or *Homo sapiens *genomes if they had a significant BLASTP hit (e-value < 0.0001) to MdBV PTP 1 (accession YP_239404). Protein sequences were aligned using MUSCLE v3.8.31 and the alignment was edited by hand in MacClade 4.06, resulting in 249 sites. The tree was built using BEAST v1.6.2 and a random local clock model for variation in the rate of evolution among branches of the tree (individual rates are estimated for each branch). Each taxon is named according to its species name and the NCBI Genbank accession number for that protein. Only posterior probability values greater than 0.7 are shown. Branches are shaded by their rate of evolution, with darker branches signifying faster rates.

Since PDVs are transmitted to offspring as integrated proviruses, the viral genome is subject to Mendelian inheritance just like the rest of the wasp nuclear genome [83,84]. This situation could have important consequences for the speed of evolution of PDV genomes relative to viruses that are transmitted horizontally. Although few estimates of mutation rates exist, analyses of four dsDNA viruses yielded mutation rates that ranged from 10^−7^ to 10^−8^ base substitutions/bp/generation, while the estimate for *Drosophila melanogaster *is one or two orders of magnitude lower, at 3.5 × 10^−9^ base substitutions/bp/generation [85,86]. BV genes also appear to be evolving faster than other genes in the wasp genome. For example, the amino acid identity of housekeeping genes in *C. congregata *and *C. inanitus *is very high (ArgK, 100%; EF1-α, 99%; Opsin, 83%; Wingless, 94%) [14]. In contrast, amino acid similarity ranges from 41-81% for the nudivirus-like genes in these two species (average 60% for 14 proteins) [36]. Gene family members present in the encapsidated genome are more divergent amongst themselves compared to their divergence from homologs in wasps and other organisms (see below). Further studies are needed to determine whether PDV genes evolve faster because of adaptive evolution or an increased mutation rate of proviral loci in the wasp genome. At the very least, however, the mutation rate is high enough to provide the raw material for the high divergence in viral genes that exist among taxa of BV-carrying wasps (see Figure 5a).

Segregation of viral core genes required for replication to locations in the wasp genome that differ from the DNAs packaged into virions could also be an important feature of PDV gene evolution. Conservation of nudivirus-like genes in braconid wasp species separated for 100 mya provides strong evidence that these genes are essential for BV virus particle production. The location of these genes within a very recombinationally active site within the proviral form of the encapsidated genome could result in loss of genes that is very detrimental for successful parasitism. However, the movement of these genes to other locations in the wasp genome potentially protects them from more frequent recombination events.

## 4. PDVs as Wasp Gene Delivery Systems

Most species of parasitic Hymenoptera do not carry PDVs but many nonetheless alter the immune defenses or growth of hosts by producing venoms, teratocytes or other sources of virulence factors. In turn, the virulence factors produced by venom glands or teratocytes are proteins encoded by wasp genes [87,88,89,90]. The novelty of PDV-carrying species is that they have also evolved a means of delivering and expressing virulence genes directly in the cells of host insects. One suggested benefit of this adaptation is it shifts the physiological cost of producing these factors from the wasp to the host [47]. Yet, there are also presumably costs to wasps in producing and packaging virions with virulence genes. In addition, all PDV-carrying wasps still produce complex mixtures of venom proteins that they inject into hosts, while all BV-carrying braconids also produce teratocytes. It would therefore seem that PDV-carrying wasps invest as much or more in the production of virulence factors as non-PDV carrying species. Thus, we think it more likely the key benefit of PDVs is it allows virulence gene products to be produced in hosts for a prolonged period [49]. This adaptation likely provides flexibility to wasps in how quickly offspring must develop, and could also allow parasitoid offspring to diapause in hosts, which increases overall development times from days to months [62]. Below, we first discuss features of PDV virions that are important for infection of host cells. We then discuss the architecture and gene content of the encapsidated genome and how selected traits potentially enhance the efficacy of PDVs as gene delivery vectors.

### 4.1. BVs Likely Use Cell Entry Mechanisms Similar to Those of Baculoviruses

Baculoviruses exhibit a complex replication cycle that involves the formation of two types of virions. The occluded form of the virus consists of enveloped nucleocapsids that are embedded in a matrix of protein called an occlusion body. Upon ingestion by a host insect, the occlusion body solubilizes in the midgut. This releases occlusion-derived virus (ODV) particles, which bind to and infect midgut cells. Replication in the midgut produces the second type of virion, named budded virus, which spreads the infection by entering other tissues of the insect’s body. The major distinguishing feature between the ODV and budded virus form is the origin and composition of the envelope that surrounds each nucleocapsid. The ODV envelope mediates binding to midgut cells and consists of several virus-encoded proteins (P74, PIF1-4, ODV-E56) that assemble in the nucleus during replication. In contrast, the envelope that surrounds budded virus nucleocapsids consists of host nuclear and/or plasma membrane components in addition to some virus-encoded proteins, some of which are also present in ODV envelopes [57,91].

Nudiviruses and BVs produce only one type of enveloped virion, and while envelope formation remains unstudied, the conserved gene set for viruses in each taxon contains homologs of the aforementioned components of ODV envelopes (see Figure 4). Electron microscopy studies of BVs likewise indicate the envelope of BV virions assembles in the nucleus during replication in a manner similar to envelope assembly for the ODV form of baculoviruses [61,64]. Given the importance of ODV envelope proteins in binding and infection of midgut cells, these findings suggest that BV homologs of these proteins are likely also critical in mediating binding and infection of host cells following parasitism by wasps. However, unlike the ODV form of baculoviruses, which more efficiently infect midgut cells than other host cell types, BV virions preferentially infect hemocytes, the fat body, and nervous system [21]. The transcriptomes of *M. demolitor*, *C. inanitus*, and *C. congregata* also contain several metalloproteinase genes that are upregulated during BV replication. Whether these metalloproteinases are constituents of BV envelopes is unclear. However, baculoviruses and nudiviruses encode metalloproteinases that are thought to be structural constituents of virus particles and to play a role in infection of host cells by digesting plasma membrane components [57,92]. Upon entry into host cells, PDV nucleocapsids traverse the cytoplasm and then discharge the viral DNA(s) into the nucleus of the host cell. Although recent studies implicate select capsid proteins of baculoviruses in actin-based motility through the cytoplasm [93], homologs of these capsid genes are absent from BVs. 

### 4.2. The Encapsidated Genomes of PDVs Have Insect-Like Architectural Features

Sequencing of several PDV isolates reveals that the architecture of the encapsidated genome differs substantially from nudiviruses, baculoviruses, and other DNA viruses. As previously noted, no DNA viruses except PDVs have segmented genomes. In addition, while most viruses have very gene-dense genomes and almost no spliced genes, the encapsidated genomes of PDVs exhibit very low coding densities and a number of genes containing introns. BV coding densities range from 17–33% and 14–69% of genes contain introns, while coding densities of nudiviruses and baculoviruses range from 67–94% and no genes have introns (Table 2). IV coding densities are similar (20–30%) to BVs, but fewer genes on average contain introns (Table 2). In contrast, the low densities of intron-containing genes of PDVs are similar to the genome architecture of hymenopterans like *Nasonia vitripennis *and *Apis mellifera* which have coding densities of 12–14% and genes that average 5 or 5.8 introns (Table 2). The G+C content of BV genomes (64–66%) is also more similar to the genomes of wasps (59–67%) than nudiviruses and baculoviruses (58–59%). 

**Table 2:** Genome-wide statistics for the AT (adenine and thymine) content, prevalence of introns, and coding density of selected BVs, IVs, and nudiviruses, baculoviruses and Hymenopterans. Taxon naming conventions are the same as for Figure 1.

Group	Genome	AT content	Introns (% of genes)	Coding density
BV genomes	CcBV	66	69	27
	CpBV	65	41	32
	CvBV	66	57	27
	GiBV	64	58	33
	GfBV	64	63	33
	MdBV	66	14	17
	CiBV	NA	NA	NA
BV relatives	HzNV-1	58	0	67
	AcMNPV	59	0	94
IV genomes				
Campoplegine IVs	CsIV	59	10	29
	HfIV	57	NA	30
	TrIV	58	NA	22
Banchine IVs	GfV	63	Near 0	20
Hymenopteran genomes	N. vitripennis	59	5 introns/gene	14
	A. mellifera	67	5.8 introns/gene	12

### 4.3. Most Genes in the Encapsidated Genome of PDVs form Multimember Families

The absence of replication genes clearly distinguishes the gene content of PDV encapsidated genomes from other viruses. A second distinguishing feature relative to other viruses is a majority of the genes they encode form families comprised of divergent members. A total of 20 gene families have been identified among BV isolates while 8 have been identified among IVs (Figure 2). Inspection of these gene families shows that most are homologs of genes found in other organisms. For example, the protein tyrosine phosphatase (PTP) gene family is present in all BV isolates studied to date except CiBV. PTPs are enzymes that regulate diverse signaling processes by dephosphorylating tyrosine residues in proteins [94]. PTPs are also found in all domains of life, with eukaryotes typically encoding multiple family members. Biochemical studies confirm that one PTP family member from MdBV is a functional enzyme, while other studies implicate the expression of BV-encoded PTPs in immunosuppression or apoptosis of host cells [76,95,96,97,98,99]. Thus like a number of pathogens, the PTP genes carried by BVs have features that indicate they function as mimics of host PTPs or pseudophosphatases to disrupt essential signaling processes. 

Comparison among isolates further shows that gene family sizes vary, and the viruses from wasp species in the same genus share more gene families with one another than viruses from wasps in more distantly related taxa (Figure 2). For example, the wasps *Cotesia congregata *and *C. vestalis *carry CcBV and CvBV respectively, which share 16/20 gene families. *C. congregata *and *Microplitis demolitor* belong to the largest subfamily in the Microgastroid complex, the Microgastrinae, but these genera are also the most distantly related to one another in this group [14]. In turn, CcBV and MdBV share only five gene families with one another and share no gene families with CiBV from *Chelonus inanitus*, which resides in the subfamily of the microgastroid complex that is most distantly related to the Microgastrinae [14] (Figure 2). The three campoplegine IV genomes that have been sequenced come from closely related wasps, and similarly encode the same gene families. In contrast, most of the gene families present in the one sequenced banchine IV genome differ from those of campoplegine IVs and BVs [29].

### 4.4. PDV Gene Families Have Diverse Origins

The preceding patterns are fully consistent with vertical transmission and Mendelian inheritance of PDVs by wasps [83,100]. However, since most genes in the encapsidated genomes of PDVs share no significant homology with genes from nudiviruses, baculoviruses, or other known viruses, they must originate from other organisms. The similarities in architecture and G+C% composition of PDV genomes relative to wasps, clearly suggest one potential source of genes in the encapsidated genome are wasps themselves. Acquisition of genes from wasps would also be consistent with the reorganization events that PDV proviral genomes have undergone (see above), and the more general trend that viruses commonly acquire genes from hosts [66,67,101]. One example of a BV gene family acquired from wasps is the sugar transporter genes found in the encapsidated genomes of *Glyptapanteles flavicoxis *bracovirus (GfBV) and *G. indiensis *BV (GiBV). These genes have complex intron/exon structure and belong to the Major Facilitator Superfamily (MFS) of transporter proteins [24]. The family is known only from BVs found in *Glyptapanteles *wasps while phylogenetic reconstruction studies indicate these genes are more closely related to orthologs in hymenopterans than other insects or other metazoans (Figure 5a). 

There are also examples of genes in PDV genomes that do not originate from wasps. The encapsidated genome of CcBV contains two copies of a *p94*-like gene known only from baculoviruses and another gene of unknown function that is similar to a gene known from ascoviruses [102]. How these genes transferred to the CcBV genome is unknown, but the evolutionary opportunity for horizontal transfer clearly exists because BV-carrying wasps are regularly exposed to baculoviruses and ascoviruses that infect the same lepidopteran hosts [103,104]. 

Lastly, several of the largest and most conserved gene families encoded by PDVs have ancient origins of uncertain history. A case in point is the previously discussed PTP family. Some baculoviruses encode PTPs but they do not share significant homology with the PTP genes from BVs. Instead, BV encoded PTPs are more similar to eukaryotic PTPs, yet the identity of the eukaryotic ancestor is unclear [25,36,76]. This is illustrated in Figure 5b which shows a phylogenetic reconstruction of the 12-member PTP family from MdBV together with PTPs from its associated wasp *M. demolitor*, two other wasp species (*A. mellifera, N. vitripennis*), and humans. This analysis strongly supports the conclusion that MdBV family members evolved by duplication from a single ancestral PTP (Figure 5b). Yet, the MdBV PTPs are equally closely related to a PTP present in each wasp species and in humans. 

The latter finding could be due to PTPs evolving at a rate that obscures their origin. Indeed, variation in rates along branches of the PTP tree indicates the evolution of the virus-encoded PTPs is much more rapid (average uncalibrated rate 1.64 units) than the rate of evolution of PTPs that reside elsewhere in the *M. demolitor *genome (average rate 0.69 units). However, Figure 5b more strongly suggests the ancestral PTP that gave rise to the viral PTP gene family either derives from a eukaryote that predates the evolution of the Hymenoptera and was lost in the three insect species sampled and humans, or the viral genome acquired the ancestral PTP by horizontal transfer from another eukaryotic genome. The analysis of PTP families from different BV genomes shows that individual family members are phylogenetically interspersed, which likewise suggests their divergence occurred before the radiation of the microgastroid complex [29,76]. Other conserved BV gene families including the ankyrins and cystatins show similar patterns of evolution [29,105], suggesting that BVs acquired several gene families early in their evolution.

### 4.5. Genome Size and Gene Content Are Highly Dynamic in PDVs

The size of individual genomic segments, the aggregate size of PDV genomes, and the number of segments per genome are extremely variable (Figure 2). For example, the encapsidated genomes of BVs from wasps in the genus *Cotesia *and *Glyptapanteles *are more than double the size of the MdBV genome from *M. demolitor*. Similar to the gene families PDVs encode, the aggregate size and number of genome segments tend to be more similar among isolates from closely related wasp species than isolates from disparate taxa. Homologous segments are also recognizable among isolates from wasps in the same genus (e.g., *G. flavicoxis *and *G. indiensis*), but due to reorganization are not recognizable at the nucleotide level when comparing segments between genera (e.g., *Glyptapanteles *and *Cotesia*) [28]. IV genomes show even greater variation in segment number as illustrated by CsIV, HfIV, and GfV. Each genome has a similar aggregate size, but CsIV has half the number of segments (24) of the HfIV genome (54), and a quarter of the number of segments present in GfV (>100) that average less than 5.2 kb in size (Figure 2). 

The genes encoded on segments are also dynamic in number. While the gene families present in closely related GiBV and GfBV genomes are very similar, the number of family members can vary substantially as exemplified by the 42 PTP genes in GiBV and 31 PTP genes in GfBV [24]). Gene families presumably expand and contract through duplication, divergence of copies (paralogs), and gene loss. Gene duplication could facilitate rapid adaptation by neofunctionalization in one paralog while purifying selection maintains the function of the other. Using sequence variation, Desjardins *et al.* (2008) looked for the signatures of selection for various genes in BVs from wasps in the genus *Glyptapanteles*. Not surprisingly, they found evidence that several gene families were evolving under positive selection [24], while studies of the cystatin gene family in BVs from wasps in the genus *Cotesia *revealed similar trends [106]. In contrast, functional studies of two structurally distinct *egf* gene family members from MdBV revealed no differences in activity as inhibitors of the host’s phenoloxidase cascade [107]. Thus, divergence of gene family members may not always result in neofunctionalization. 

One advantage of a segmented genome is it provides flexibility in terms of the type and number of genes that PDVs can deliver to hosts. Another is that it provides a mechanism for adjusting gene dosage in the absence of replication. Specifically, the genome segments of PDVs are not equimolar in abundance. Since BVs package only one genomic segment per virion, this means that virions containing certain segments are more abundant than others in the mixture of virus particles that a wasp injects into a host [16,21]. Experimental studies show that wasps inject enough virions to infect each immune cell of the host with the entirety of the genome. However, on average immune cells contain higher copy numbers of more abundant genomic segments than less abundant segments. In turn, transcript abundance of genes on more abundant segments tends to be higher than for genes on less abundant segments [21]. Segment nesting by IVs is thought to also be an adaptation for adjusting gene dosage [7]. Whether fine-tuning of segment abundance occurs among PDV isolates is unknown. Yet, such compensatory responses are likely given that most PDV-carrying wasps have evolved to parasitize different species of hosts.

Less clear is the processes by which genomic segments and genes move in PDV genomes. In CcBV, gene duplication of single genes or blocks of genes is often followed by rapid movement of one duplicate copy onto another BV genome segment, suggesting frequent shuffling of genes in the genome [108]. One mechanism for movement of segments is recombination, presumably between homologous sequences at unlinked sites in the genome [108,109]. Another may be through the activity of transposable elements. Several such elements are present in BV genomes, including a transposon similar to *p−-*element in GiBV, and DIRS and Dong-like elements in CcBV [24,102]. In GfBV, Gypsy, *Mariner*-like and *Maverick*-like transposable elements are located in a sequence abutting a proviral segment [110]. The presence of a *ptp* gene in this proviral flanking sequence identical to a *ptp* within the neighboring proviral segment further suggests one of the neighboring transposable elements facilitated this duplication [24]. Transposases could also contribute to gene movement by facilitating the integration of cDNAs into the proviral genome. Two potential examples of this are BV cystatins and IV innexins, which lack the introns present in insect genes of the same function suggesting they arose from reverse-transcribed insect genes [25,80,111]. Lastly, transposable element genes may be co-opted into PDV genomes as in the case of an aspartyl protease gene from *Tn*BV that shows signatures of a retroviral origin [112].

### 4.6. DNA Segments of the Encapsidated Genomes Integrate into Host Cells

A final feature of PDV genomes important for gene delivery to hosts is the finding that viral DNAs rapidly integrate into the genome of host cells [62]. Early studies suggested that PDVs likely persist in the nuclei of host cells as episomes, which is fully adequate for the transient expression of immunosuppressive and other virulence genes. However, the long-term detection of some PDV genomic DNAs in cell lines established from lepidopteran hosts [113] suggested the possibility that PDV DNAs may be capable of integrating. Unclear from these cell culture studies though is whether PDVs actually integrate into hosts during parasitism. Recent studies with MdBV indicate the answer to this question is yes, and that the viral genes essential for immunosuppression continue to be expressed after integration [62]. Interestingly, the integration of individual genomic segments appears to be site specific into the genome of host cells and is mediated by a conserved direct repeat element that differs from the flanking motifs that identify the sites of segment integration in the genome of the wasp [62]. The mechanism of integration remains unknown although features suggest it occurs by non-homologous recombination and depends in part on the integration machinery of the host. The ability of segments to integrate into host cells explains how long-term expression of PDV gene products occurs in hosts [62], and shed light into how the offspring of some wasp species could evolve to overwinter by diapausing in host larvae.

## 5. Concluding Remarks

Viruses are usually thought of as parasitic entities that adversely affect or kill their hosts. However, the association of PDVs with wasps clearly shows that viruses also form symbiotic associations. The larger question is whether PDVs are a rare example of virus-host symbiosis, or might the evolution of symbioses between viruses and other organisms be more common than the literature suggests? We think the latter more likely given that other examples exist where viruses have established associations of benefit to another organism. In insects, the phage APSE infects the facultative bacterial symbiont *Hamiltonella defensa* of aphids, and encodes genes implicated in defense of aphids against aphidiine parasitoid wasps [114,115,116]. The WO phage of bacteria has also been implicated in affecting the reproductive interactions between its hosts, intracellular bacteria in the genus *Wolbachia*, and wasps like *Nasonia vitripennis, *which is a host for the bacterium [117]. Combined with increasing recognition that many viruses establish persistent infections [59,65,118,119,120,121,122,123,124], the evolutionary opportunity for symbiotic associations is large. Future studies will undoubtedly identify other examples of virus-host symbiosis to complement the elegant relationship that exists between PDVs and wasps.
